# Comparative Analysis for the Performance of Variant Calling Pipelines on Detecting the *de novo* Mutations in Humans

**DOI:** 10.3389/fphar.2019.00358

**Published:** 2019-04-11

**Authors:** Yu Liang, Li He, Yiru Zhao, Yinyi Hao, Yifan Zhou, Menglong Li, Chuan Li, Xuemei Pu, Zhining Wen

**Affiliations:** ^1^College of Chemistry, Sichuan University, Chengdu, China; ^2^Biogas Appliance Quality Supervision and Inspection Center, Biogas Institute of Ministry of Agriculture, Chengdu, China; ^3^College of Computer Science, Sichuan University, Chengdu, China

**Keywords:** *de novo* mutation, rare diseases, variant calling pipelines evaluation, gene function, whole-exon sequencing

## Abstract

Despite of the low occurrence rate in the entire genomes, *de novo* mutation is proved to be deleterious and will lead to severe genetic diseases via impacting on the gene function. Considering the fact that the traditional family based linkage approaches and the genome-wide association studies are unsuitable for identifying the *de novo* mutations, in recent years, several pipelines have been proposed to detect them based on the whole-genome or whole-exome sequencing data and were used for calling them in the rare diseases. However, how the performance of these variant calling pipelines on detecting the *de novo* mutations is still unexplored. For the purpose of facilitating the appropriate choice of the pipelines and reducing the false positive rate, in this study, we thoroughly evaluated the performance of the commonly used trio calling methods on the detection of the *de novo* single-nucleotide variants (DNSNVs) by conducting a comparative analysis for the calling results. Our results exhibited that different pipelines have a specific tendency to detect the DNSNVs in the genomic regions with different GC contents. Additionally, to refine the calling results for a single pipeline, our proposed filter achieved satisfied results, indicating that the read coverage at the mutation positions can be used as an effective index to identify the high-confidence DNSNVs. Our findings should be good support for the committees to choose an appropriate way to explore the *de novo* mutations for the rare diseases.

## Introduction

The genomic structural variations, such as single-nucleotide variants (SNVs), copy-number variants (CNV) and the indels, play important roles in the genetic diseases. The researches in the past decade have discovered the landscape of SNVs in human and the strong causality between the SNVs and the genetic diseases (Ku et al., 2012; Veltman and Brunner, 2012; Boycott et al., 2013). Among the SNVs, the occurrence frequency of *de novo* SNVs (DNSNVs) in germline is as low as 1.0∼3.0 × 10^−8^ SNVs per site per generation (Conrad et al., 2012; Veltman and Brunner, 2012), but this type of mutation is proved to be deleterious and will lead to severe genetic diseases via impacting on the different gene functions. It had been reported in recent studies that the rare sporadic malformation syndromes (Hoischen et al., 2010a,b; Ng et al., 2010) as well as the neurodevelopmental diseases (Hamdan et al., 2014; Turner et al., 2017) were primarily caused by the DNSNVs in single specific genes or a set of genes, elaborating the fundamentality of the *de novo* mutations in the genetic diseases despite of the unclear underlying mechanisms. Therefore, accurately identifying the *de novo* mutations located in the rare-disease-causing genes can be great helpful not only for improving the clinical diagnostics, but also for better understanding the mechanisms in the rare genetic diseases.

Due to the fact that the traditional family based linkage approaches and the genome-wide association studies were unsuited to the detection of *de novo* mutations, the emerging next-generation sequencing technologies such as the whole-genomes sequencing (WGS)/whole-exome sequencing (WES) began to be applied in the researches of genetic diseases (Boycott et al., 2013; Peters et al., 2015; Jin et al., 2017; Turner et al., 2017; Hyrenius-Wittsten et al., 2018). A number of bioinformatics pipelines have subsequently been proposed to call the *de novo* mutations based on the WGS/WES data (McKenna et al., 2010; Li et al., 2012; Koboldt et al., 2013; Kojima et al., 2013; Peng et al., 2013; Ramu et al., 2013; Cleary et al., 2014; Salzberg et al., 2014; Santoni et al., 2014; He et al., 2015; Wei et al., 2015; Francioli et al., 2017; Gomez-Romero et al., 2018; Zhou et al., 2018). The heated discussions have been carried on in recent years about applying these approaches in the diagnostics of rare diseases and the potential clinical implementations (Yang et al., 2013; Lee et al., 2014; Jamuar and Tan, 2015; Bacchelli and Williams, 2016; Krier et al., 2016; Thiffault and Lantos, 2016). However, the occurrence of germline *de novo* mutations is much lower than that of the inherited variations, resulting in the difficulty of discriminating these variants from the noise derived from the procedures of sequencing, reads mapping as well as the variant calling and annotation. So far how the performance of these variant calling pipelines on detecting the *de novo* mutations is still unexplored.

Therefore, in our study, we thoroughly investigated three commonly used trio calling pipelines named GATK (McKenna et al., 2010; Francioli et al., 2017), RTG (Cleary et al., 2014) and VarScan (Koboldt et al., 2013) on the detection of the DNSNVs and found that GATK can detect the DNSNVs in the low GC-content region with a relative low error rate while RTG and VarScan are more suitable for detecting the DNSNVs in the high GC-content region. In refining the calling results of a single pipeline, our proposed filter not only effectively excluded the redundant DNSNVs, but also ensured the transitions/transversions ratio of the results, indicating that the read coverage at the mutation positions of the son’s genome and the parents’ genomes can be an important index for evaluating the quality of DNSNVs.

## Materials and Methods

### Dataset

The WES data of the Ashkenazi Jewish (AJ) trio set (NA12878) were applied in our study (Zook et al., 2014, 2016). The preprocessed BAM files of the mother (HG004), the father (HG003) and the son (HG002), for which the data preprocessing steps including the reads alignment and duplicates marking had been conducted beforehand, were directly downloaded from the Genome in a Bottle (GIAB) website^1^ and used for calling the *de novo* mutations. In order to facilitate the comparison of the similarities and differences of the pipelines and make the point clear, in our study, we only focused on the *de novo* SNVs on the autosomes. It is worth noting that the structure variations, e.g., indels, as well as the mutations on the chromosomes X and Y are also important for the genetic diseases, but it will make the problem more complicated when involving them in the comparisons.

### Trio Calling Pipelines

Three pipelines, namely GenomeAnalysisToolKit (version 4.0.5.2) (McKenna et al., 2010; Francioli et al., 2017), RTG (non-commercial version 3.9.1) (Cleary et al., 2014) and VarScan (version 2.3.9) (Koboldt et al., 2013), were applied in this study to call the DNSNVs. In GATK pipeline, the gVCF files for the trio samples were firstly generated by using *HaplotypeCaller* separately and combined into a multi-sample gVCF file through *CombineGVCFs*. Then, the raw SNVs were called by *GenotypeGVCFs* and recalibrated by *VariantRecalibrator* and *ApplyVQSR*. Finally, after deriving the posterior probabilities of the genotypes by *CalculateGenotypePosteriors*, the low quality genotypes were filtered out by *VariantFiltration* and the *de novo* SNVs were annotated by *VariantAnnotator*. In RTG pipeline, the quality calibration files for the BAM files of trio samples were firstly prepared and applied in the subsequent calling procedures. The *de novo* SNVs were called and extracted by RTG with two parameters of *family* and *vcffilter*. As to the Varscan pipeline, the BAM files of trio samples were firstly sorted and combined into a three-sample pileup file. Subsequently, the *de novo* SNVs were called by running the *VarScan trio* command.

### Metrics for the Comparison of the Pipelines

We used three metrics namely GC content (Shin et al., 2013), substitution type (Zook et al., 2014) and SNV density (Choi et al., 2018), to evaluate the differences in the calling results generated by three pipelines. A genomic sequence of 100 bases centered on a DNSNV was extracted from the reference genome and the GC content can be calculated via the following equ.

(1) $$\mathrm{GC content}\;(\%)=\frac{\mathrm{number of bases G and C}}{100}\times 100\%$$

The substitution type includes the point mutations of transition and transversion. The transition refers to the nucleotide changes from a purine to another purine (A↔G) or a pyrimidine to another pyrimidine (C↔T), which occurs more frequently than the transversion in the SNPs. The transversion refers to the changes from purine to pyrimidine, or vice versa (A↔T, A↔C, G↔T, and C↔C). By counting the total number of SNVs in the genomic sequence of 100 bases centered on a DNSNV, the SNV density can be calculated via the equ. (2):

(2) $$\mathrm{SNV density}\;(\%)=\frac{\mathrm{number of SNVs}}{100}\times 100\%$$

### Definition of DNSNV Filter

To distinguish the high-confidence DNSNVs from the noise, the read coverage at the mutation point is an important indicator for this purpose. Based on the concept of signal-to-noise ratio, we not only expect that the number of reads mapped to the reference genome at the mutation point for the parents’ datasets is high than that mapped to the mutated sequence as much as possible, but also expect that the number of reads mapped to the mutated sequence for the son’s dataset is higher than that mapped to the reference genome. Therefore, to refine the calling results from the pipelines, we proposed a DNSNV filter by considering both read coverage at the mutation point in the son’s dataset and the parents’ datasets. The filter was defined as the following equ:

(3) $$\text{Score}=\text{log}\left(\frac{\left|{\text{F}}_{\text{ref}}-{\text{F}}_{\text{alt}}|+|{\text{M}}_{\text{ref}}-{\text{M}}_{\text{alt}}\right|}{2\times |{\text{S}}_{\text{alt}}-{\text{S}}_{\text{ref}}|}\right)$$

Where the F_ref_, M_ref,_ and S_ref_ indicate the number of reads mapped to the reference genome at the ith mutation point for the datasets of father, mother and son, respectively. The F_alt_, M_alt,_ and S_alt_ indicate the number of reads mapped to the mutated sequence at the *i*th mutation point for the datasets of father, mother and son, respectively. We assigned the scores for the DNSNVs and rank them by their scores. In this study, we took a non-stringent score (score = 0) as the threshold to investigate the improvement of the calling results.

### Genotype Quality

Genotype Quality (GQ) (Zhang et al., 2013) is used to evaluate the filtering results of DNSNVs, which indicates the quality value of the most likely genotype. The quality value refers the possibility of the genotype being present at the site. The larger value means the greater the likelihood of the genotype.

## Results

To facilitate the appropriate choice of the trio calling pipelines for detecting the DNSNVs, in our study, we firstly evaluated the results of three commonly used pipelines named GATK, RTG, and VarScan by using the WES data of a father-mother-child trio set from Ashkenazi Jews, and then proposed a filter for removing the redundant DNSNVs from the calling results.

### The Landscape of the Calling Results From Three Pipelines

**FIGURE 1:**
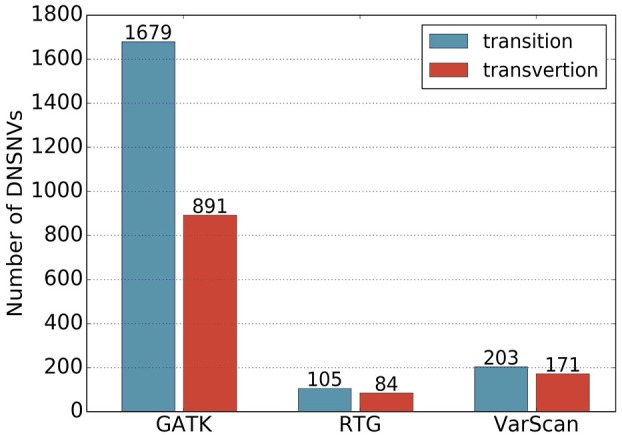
The numbers of transitions and transversions in the calling results generated by GATK, RTG and VarScan.

In total, 2570, 189 and 374 DNSNVs in the autosomes were identified by GATK, RTG and VarScan, respectively. In terms of the quantity of DNSNVs, GATK exhibited higher detecting sensitivity than RTG and VarScan. For the point of mutation types, Figure 1 showed clear difference between the numbers of transitions and transversions in the calling results of three pipelines. Generally the number of transitions was greater than that of the transversions for all the pipelines. The transition/transversion ratio (Ti/Tv) had been suggested in GIAB study (Zook et al., 2014) to be a metric for evaluating the quality of the novel calling variants based on the assumption that Ti/Tv in novel variants would be similar to that in the common variants. In our results, Ti/Tv for GATK was 1.88 (1679/891), which was higher than those for the other two pipelines [1.25 (105/84) and 1.19 (203/171) for RTG and VarScan, respectively], indicating a lower error rate in the GATK calling results. In addition, we mapped the DNSNVs to the dbSNP database^2^ and separately calculated the overlap rates between the DNSNVs and all the variations, as well as between the DNSNVs and the common variations for the three pipelines (Supplementary Figure S1). The overlap rate was calculated by dividing the number of overlapped DNSNVs by the total number of DNSNVs identified by the pipeline. From the figure we can see that GATK achieved the highest overlap rate among three pipelines, indicating the least proportion of DNSNVs involved in the calling results. On the contrary, VarScan achieved the lowest overlap rate, indicating the largest proportion of DNSNVs involved in the calling results. Meanwhile, the Ti/Tv rates of three pipelines indicated the lowest error rate in the calling results of GATK and highest error rate in the results of VarScan. It suggested that VarScan and RTG tend to reveal more DNSNVs in their reported results, but the reliability of them needs to be more carefully validated by further experiments.

**FIGURE 2:**
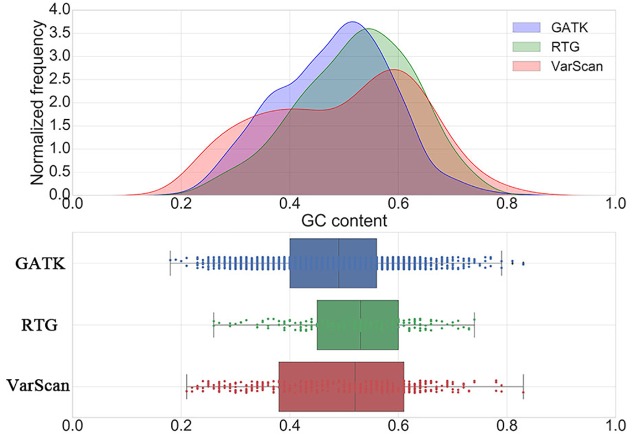
The distribution of GC contents around the *de novo* SNVs identified by GATK, RTG and VarScan.

Considering the fact that with the increase of GC content, the difficulty of polymerase chain reaction (PCR) amplification in sequencing procedure will increase, which will result in an increase in the error rate of the calling results, we further investigated the distribution of GC contents around the DNSNVs for three pipelines (Figure 2). The majority of the DNSNVs called by GATK located in the regions with GC contents less than 50% while the DNSNVs called by RTG mainly located in the regions with relatively high GC contents (>50%). This may be the reason that GATK can yield the higher Ti/Tv ratio (1.88) than that (1.25) obtained by RTG. Interestingly, although VarScan yielded a relatively low Ti/Tv ratio (1.19), the distribution of the calling results from VarScan had two peaks that covered the regions with both low (∼40%) and high (∼60%) GC content. We separately inspected the point mutation types of the DNSNVs called by VarScan in the genome regions with GC content ≥50% and GC content <50%. The Ti/Tv ratios for the high (≥50%) and low (<50%) GC-content regions were 1.32 (116/88) and 1.05 (87/83), respectively. It may indicate that, for the high GC-content region, the VarScan can identify the DNSNVs with even lower error rate than RTG (Ti/Tv = 1.25). As to calling the DNSNVs in the low GC-content region, the performance of VarScan (Ti/Tv = 1.05) is inferior to GATK (Ti/Tv = 1.88).

**FIGURE 3:**
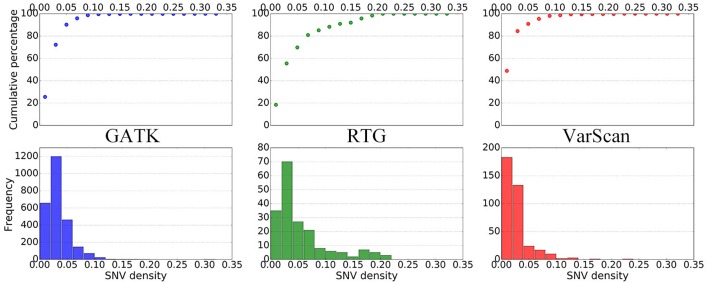
The distribution of SNV densities around the *de novo* SNVs identified by GATK, RTG, and VarScan.

To further elucidate the performance of the three pipelines, we subsequently summarized the distributions of SNV densities around the DNSNVs (Figure 3). As the SNV density increases, the error rate for calling DNSNVs may increase. It can be seen from the figure that, for the RTG pipeline, only ∼70% DNSNVs located in the regions with the SNV density less than 5%, and the accumulated percentage gradually increased to 90% when counting the DNSNVs in the regions with the SNV density less than 15%, indicating a larger error rate may exist in the call results. Compared to RTG, over 90% DNSNVs called by GATK and VarScan located in the regions with the SNV density less than 5%, which may suggest the better quality of the identified DNSNVs.

### Performance of Our Proposed Filter on Refining the DNSNVs

**FIGURE 4:**
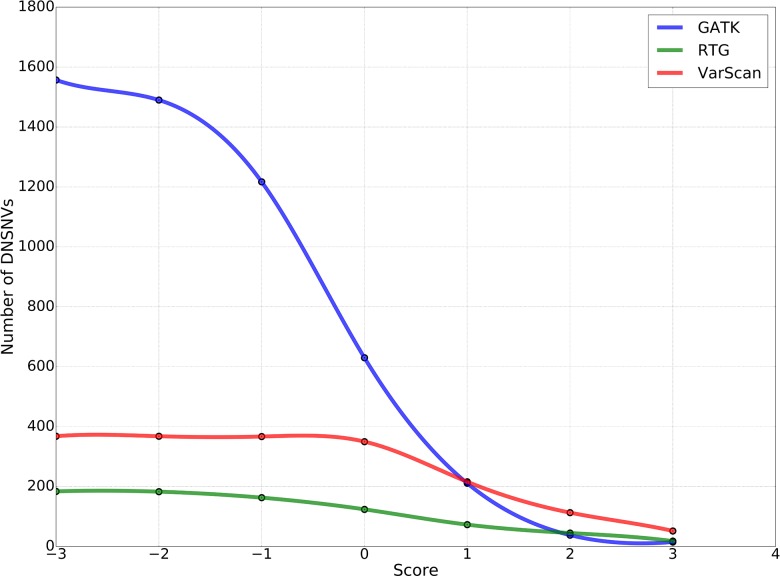
The number of *de novo* SNVs kept in the calling results when filtered by using different cut-offs in our proposed filter.

To obtain the high-confidence DNSNVs, we suggested further refining the calling results of three pipelines by using a proposed filter, which was taken both read coverage at the mutation sites of the son’s genome and the parents’ genomes into consideration to identify the DNSNVs and can be directly applied to an individual pipeline without reference to the information of other pipelines. Figure 4 showed the numbers of DNSNVs kept by the filter when applying different cut-offs. The number of DNSNVs called by all the pipelines took on a tendency of descension when the cut-off became more stringent, especially for the GATK pipeline. The number of the left DNSNVs called by GATK dramatically decreased from over 1,500 to 15 as the value of the cut-off increased from −3 to 3.

**FIGURE 5:**
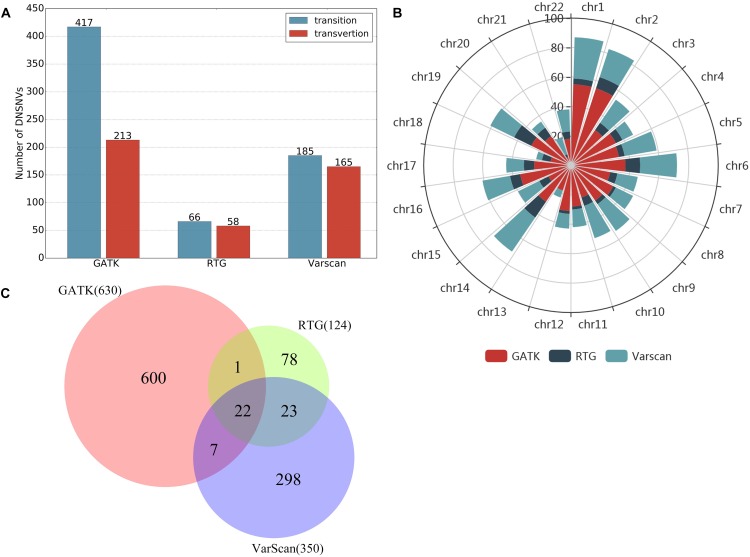
Results on the filtered *de novo* SNVs identified by GATK, RTG and VarScan. **(A)** The number of transitions and transversions in the filtered calling results. **(B)** The chromosomal distribution of the filtered *de novo* SNVs. **(C)** The overlaps of the filtered *de novo* SNVs among three pipelines.

In this study, we just took a non-stringent cut-off (score = 0) as an example for the comparison of the three pipelines. When filtering the DNSNVs with the score >0, the numbers of DNSNVs detected by GATK decreased from 2570 to 630. For RTG and VarScan, the numbers of DNSNVs decreased from 189 and 374 to 124 and 350, respectively. Figure 5A showed the number of transitions and transversions in the filtered calling results. The GATK pipeline still yielded the highest ratio (Ti/Tv = 1.96) among three pipelines, which was improved after filtering a number of DNSNVs from the calling results. For the pipelines of RTG and VarScan, since only a small number of DNSNVs were removed, the quality of final DNSNVs were comparable to that of the original calling results, with a slight decrease in the ratio (Ti/Tv = 1.14 and 1.12 for RTG and VarScan, respectively). Our results may suggest that our proposed filter can efficiently reduce the error rate in the DNSNVs from the redundant calling results. In addition, for the calling results with less redundancy, our filter can well maintain the quality of the original results.

The chromosomal distribution of the filtered DNSNVs was shown in Figure 5B. The DNSNVs identified by three calling pipelines mainly located on the chromosomes 1, 2, 6, 14, 16, and 19, which indicates that the DNSNVs are more likely to occur on these six chromosomes. Conversely, the DNSNVs rarely occur on chromosomes 13, 18, and 21. Figure 5C showed the overlaps of the filtered DNSNVs among three pipelines. Because both RTG and VarScan were adapted for detecting the DNSNVs in the high GC-content region with relative low error rate, close to 36% (45/124) DNSNVs identified by RTG can be found in the results called by VarScan even if 65 out of 189 DNSNVs identified by RTG had been removed out by the filter. When comparing GATK with RTG and VarScan, only about 19% (23/124) and 8% (29/350) DNSNVs called by RTG and VarScan, respectively, were involved in the calling results of GATK. The reason may be that GATK was fit for calling the DNSNVs in the low GC-content region while the ability of RTG and VarScan to identify the DNSNVs in this region is inferior. This should be further validated by using the real disease samples. Eventually, a total of 22 DNSNVs were detected by all the pipelines.

**FIGURE 6:**
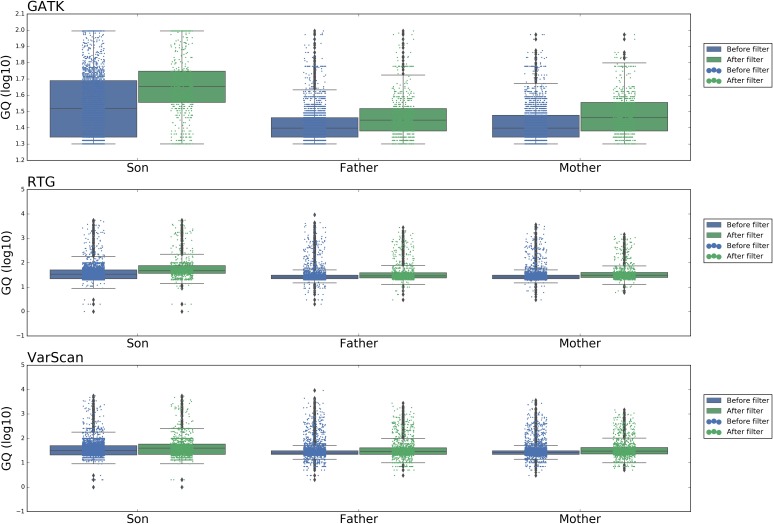
The distribution of GQ around the *de novo* SNVs identified by GATK, RTG, and VarScan.

Figure 6 showed the distribution of DNSNVs GQ of the trio before and after filtering. For GATK, significant difference exists between the calling results before and after filtering. However, for RTG and VarScan, the difference is slight. But for the latter two pipelines, the filtered DNSNVs GQ distribution is still slightly better than that before filtering. In general, the GQ distributions of DNSNVs for the three pipelines after filtering are better than those before filtering.

### Biological Relevance of the Overlapped DNSNVs Among Three Pipelines

The 22 overlapped DNSNVs can be detected by three pipelines simultaneously, and should be of relatively high confidence. To investigate the biological relevance of these DNSNVs, we firstly mapped them to the corresponding genes and then explored the associations of the genes with genetic diseases by the Online Mendelian Inheritance in Man (OMIM) database searching and literature survey. The description of 22 DNSNVs as well as the corresponding genes finally identified by all pipelines was listed in Table 1. In the results, six out of 22 genes has been proved to be directly associated with the rare genetic diseases. For example, the gene *DTNB* in chromosome 2 is an important protein-coding gene of beta-dystrobrevin. This protein is found to interact directly with dystrophin and the low expression level of it will cause severe Duchenne muscular dystrophy (Blake et al., 1998). The genes *FIG4* and *LAMC3* were reported to be highly correlated with the polymicrogyria and cortical malformations, respectively. Campeau et al. (2013) demonstrated that the inactivation of *FIG4* would result in the central nervous system dysfunction and extensive skeletal anomalies. The research by Barak et al. (2011) exhibited an important role of the gene *LAMC3* in cortical organization. It can be seen that to a certain extent our proposed filter can be helpful for removing the redundant DNSNVs from the calling results.

**Table 1:** The 22 overlapped DNSNVs identified by all the trio calling methods and the corresponding genes associated with the diseases.

Chromosome	Position	Substitution	Gene symbol	phenotype
2	25457155	C→T	DTNB^∗^	Muscular dystrophy
2	197791183	C→T	–	–
2	241835203	G→T	–	–
3	4669342	A→T	ITPR1^∗^	Gillespie syndrome Spinocerebellar ataxia 15 Spinocerebellar ataxia 29, congenital non-progressive
3	48603870	G→A	UQCRC1	Predisposition of Alzheimer’s disease
3	52547912	C→G	PBRM1^∗^	Clear cell renal cell carcinoma
6	109740508	T→ C	FIG4^∗^	Polymicrogyria, bilateral temporooccipital Amyotrophic lateral sclerosis 11 Charcot-Marie-Tooth disease, type 4J Yunis-Varon syndrome
5	140730969	A→G	–	–
8	30703949	C→G	GSR^∗^	Hemolytic anemia due to glutathione reductase deficiency
9	131041047	G→T	LAMC3^∗^	Cortical malformations, occipital
9	131851319	C→G	–	–
11	82698724	T→C	–	–
14	77245340	C→T	TMEM63C	
14	93170535	G→ A	–	–
14	96180196	C→G	–	–
14	106330036	T→A	–	–
14	106993919	A→G	–	–
15	79852462	G→T	MTHFS	Heart defects; lung cancer
17	53638886	G→A	–	–
19	45720935	C→T	FBXO46	–
22	23029544	A→C	–	–
22	23029596	A→T	–	–

*^∗^The genes were reported to be directly associated with genetic diseases in the OMIM database.*

## Discussion

Nowadays, it has been proved that the *de novo* mutations played an important role in human genetic diseases. Based on the next-generation sequencing technology, more and more researches focused on detecting the *de novo* mutations in the rare genetic diseases as well as the potential applications in the clinics for the purpose of improving the clinical diagnosis and better understanding the mechanisms of the genetic diseases. However, it is still a tough work and remains challenging to accurately identify the genomic variants because of the complexity of the sequencing experiments and the variants calling procedures. In the previous study, Reumers et al. (2012) suggested that an optimized filtering procedure would be helpful for reducing the error rate when detecting the genomic variants with the short-read sequencing data. Considering the fact that the occurrence rate of *de novo* mutations is much lower than those of inherited variations and somatic variants, it is more difficult to distinguish them from the errors in the whole genome. At present, a number of trio calling methods were proposed to detect the *de novo* mutations based on the WGS/WES data, but how the performance of these pipelines on detecting the *de novo* mutations is still unexplored. Therefore, by carefully comparing the results from three commonly used trio calling pipelines, we elucidated that the performance of the three pipelines on calling DNSNVs in high or low GC-content region was different. In addition, based on the read coverage, our proposed filter can be well applied to the single pipeline for refining the calling results.

In this study, we analyzed the calling results from three pipelines named GATK, RTG and VarScan. Generally speaking, GATK can identify the DNSNVs in the low GC-content region with the lowest error rate among the three pipelines while RTG tends to detect the DNSNVs in the high GC-content region. Considering the effect of high GC-content on experimental and computational results of SNV detection, the Ti/Tv ratio achieved by RTG was lower than that achieved by GATK, indicating a higher error rate in RTG calling results. Therefore, when using single pipeline to identify the DNSNVs, people should pay more attention to the DNSNVs in the GATK calling results that fell into the high GC-content region, or the DNSNVs in the RTG results that fell into the low GC-content region. For VarScan, although the calling results covered a broad region of the GC-content, the Ti/Tv ratio of the DNSNVs in the low GC-content region was only 1.05, which is much lower than that achieved by GATK (Ti/Tv = 1.88). The Ti/Tv ratio of the DNSNVs in the high GC-content region (Ti/Tv = 1.32) was comparable to that achieved by RTG (Ti/Tv = 1.25). So, people still need to carefully validate the DNSNVs detected in the low GC-content region when using VarScan for calling.

For the purpose of removing the redundant DNSNVs, we proposed a filter to refine the calling results for the single pipeline by considering the read coverage at the mutation sites of the son’s genome and the parents’ genomes. Our results showed that a number of DNSNVs were removed from the GATK calling results when applying a non-stringent cut-off (score = 0) and the Ti/Tv ratio of the left DNSNVs increased, indicating an improvement of the calling results. For the less redundant results, e.g., the DNSNVs detected by VarScan, only a small number of DNSNVs were filtered out and the Ti/Tv ratio did not changed significantly. Our findings indicated that the proposed filter might be benefit to the refinement of the DNSNVs identified by current pipelines.

It is worth noting that, considering the fact that the size of the input data is small, the filtering algorithm can be further improved by giving a confidence index score and a statistical test index, (e.g., *p*-value) for the score of a DNSNV, which can be estimated from the mapping probability of the reads and the confidence level of the read coverage. It is helpful for increasing the confidence level of the findings. Moreover, due to the limitation of samples, we only used normal samples for the comparative analysis in this study. The work could be further improved by using genetic disease samples, which would make the evaluation of the variant calling error rate of the specific pipeline more accurate. When constructing the filtering algorithm, we only used the exome sequence as an input. The proposed algorithm can be broadened to the analysis of whole genome sequence by integrating additional steps to trim the whole genome. Additionally, we only discussed the difference of *de novo* SNVs detected by different pipelines. In fact, other types of *de novo* structural variations, such as indels, also play important roles in the biological processes in the genetic diseases. While taking more types of structural variations into account will make the comparative analysis more complex, it does contribute to a more comprehensive assessment of the performance of existing pipelines. These issues will be further addressed in our future study.

## Conclusion

In this study, we demonstrated that different pipelines have a specific tendency to detect the DNSNVs in the genomic regions with different GC contents. GATK performed better on detecting the DNSNVs in the low GC-content region while RTG and VarScan are better suited for detecting the DNSNVs in the high GC-content region. To refine the calling results for single pipeline, the read coverage at the mutation positions of the son’s genome and the parents’ genomes can be considered as an effective index to identify DNSNVs with high confidence. Our findings would be useful for the community to choose the appropriate pipelines and obtain the calling results with high confidence when discovering the *de novo* mutations for the genetic diseases.

## Author Contributions

ZW designed the experiments. YL, LH, YZ, and YH performed the data analysis. ZW wrote the initial version of manuscript. YZ, YL, and ML prepared all the figures. ZW, CL, and XP discussed the results and revised the manuscript. All authors contributed to discussions regarding the results and the manuscript.

## Conflict of Interest Statement

The authors declare that the research was conducted in the absence of any commercial or financial relationships that could be construed as a potential conflict of interest.

## Footnotes

1 ftp://ftp-trace.ncbi.nlm.nih.gov/giab/ftp/data/AshkenazimTrio/

2 https://www.ncbi.nlm.nih.gov/snp/

## Supplementary Material

FIGURE S1 The overlap rates between the DNSNVs and the variants in the dbSNP database.

## Abbreviations

AJ: ashkenazi jewish
CNV: copy-number variants
DNSNVs: *de novo* single-nucleotide variants
GATK: genomeanalysistoolkit
GIAB: genome in a bottle
OMIM: online mendelian inheritance in man
PCR: polymerase chain reaction
SNVs: single-nucleotide variants
Ti/Tv: the transition/transversion ratio
WES: whole-exome sequencing
WGS: whole-genomes sequencing.
