# Supplementary figures and images for: Comparative Analysis for the Performance of Variant Calling Pipelines on Detecting the de novo Mutations in Humans
Source: Front Pharmacol. 2019 Apr 11;10:358. doi: 10.3389/fphar.2019.00358 (PMC6499170; doi:10.3389/fphar.2019.00358)

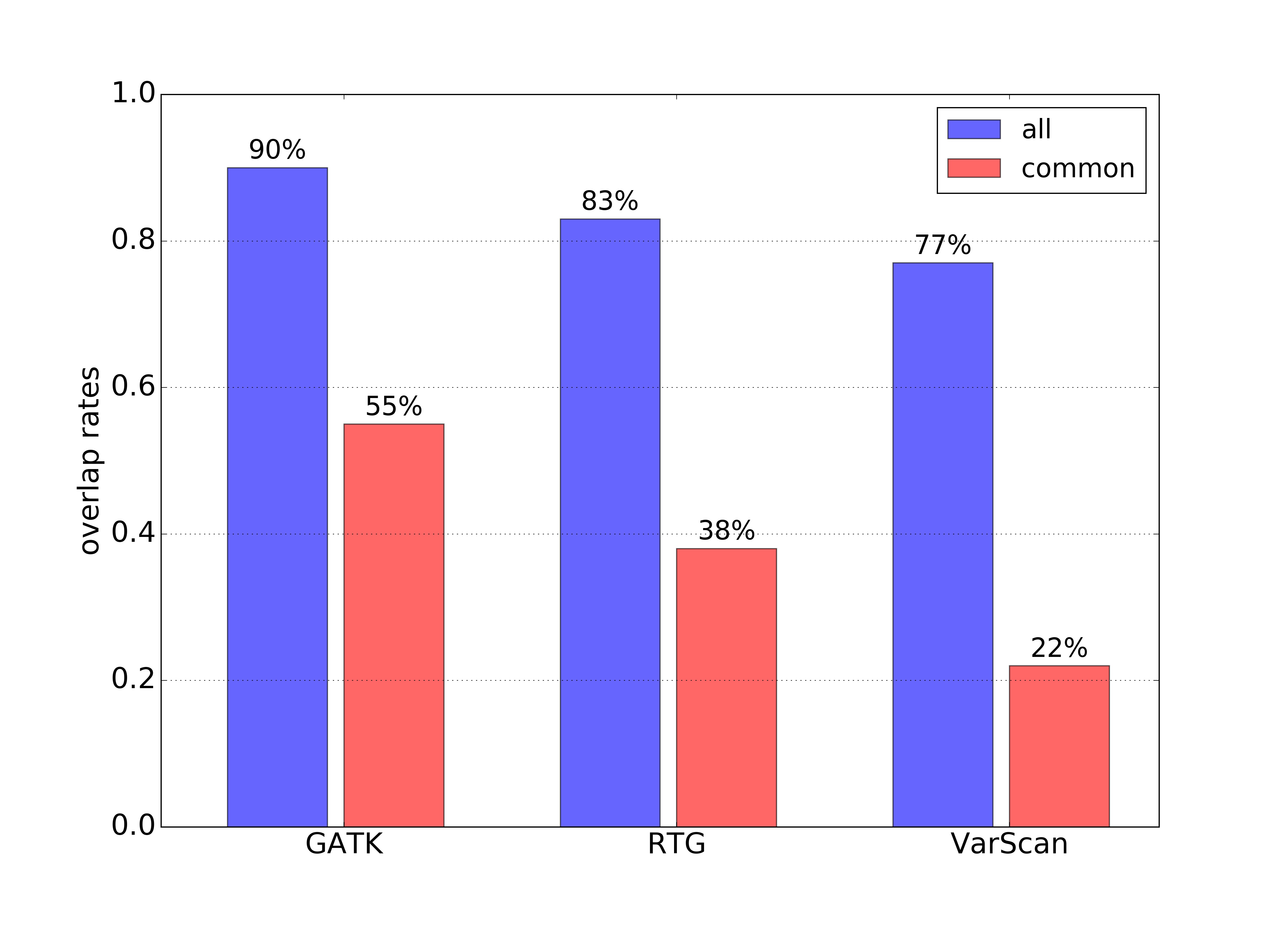

Supplement: FIGURE S1 — The overlap rates between the DNSNVs and the variants in the dbSNP database. [file Image_1.jpg]
